# Interruption of persistent exposure to leprosy combined or not with recent BCG vaccination enhances the response to *Mycobacterium leprae* specific antigens

**DOI:** 10.1371/journal.pntd.0005560

**Published:** 2017-05-03

**Authors:** Fernanda Marques de Carvalho, Luciana Silva Rodrigues, Nádia Cristina Duppre, Iris Maria Peixoto Alvim, Marcelo Ribeiro-Alves, Roberta Olmo Pinheiro, Euzenir Nunes Sarno, Maria Cristina Vidal Pessolani, Geraldo Moura Batista Pereira

**Affiliations:** 1 Laboratory of Cellular Microbiology, Oswaldo Cruz Institute, FIOCRUZ, Rio de Janeiro, Brazil; 2 Leprosy Laboratory, Oswaldo Cruz Institute, FIOCRUZ, Rio de Janeiro, Brazil; 3 Laboratory of Clinical Research in DST- AIDS, Institute of Clinical Research Evandro Chagas, FIOCRUZ, Rio de Janeiro, Rio de Janeiro, Brazil; Johns Hopkins Bloomberg School of Public Health, UNITED STATES

## Abstract

Household contacts of multibacillary leprosy patients (HCMB) constitute the group of individuals at the highest risk of developing leprosy. Early diagnosis and treatment of their index cases combined with Bacille Calmette-Guerin (BCG) immunization remain important strategies adopted in Brazil to prevent HCMB from evolving into active disease. In the present study, we assessed the impact of these measures on the immune response to *Mycobacterium leprae* in HCMB. Peripheral blood mononuclear cells (PBMC) from HCMB (n = 16) were obtained at the beginning of leprosy index case treatment (T0). At this time point, contacts were vaccinated (n = 13) or not (n = 3) in accordance with their infancy history of BCG vaccination and PBMCs were recollected at least 6 months later (T1). As expected, a significant increase in memory CD4 and CD8 T cell frequencies responsive to *M*. *leprae* whole-cell sonicate was observed in most contacts. Of note, higher frequencies of CD4^+^ T cells that recognize *M*. *leprae* specific epitopes were also detected. Moreover, increased production of the inflammatory mediators IL1-β, IL-6, IL-17, TNF, IFN-γ, MIP1-β, and MCP-1 was found at T1. Interestingly, the increment in these parameters was observed even in those contacts that were not BCG vaccinated at T0. This result reinforces the hypothesis that the continuous exposure of HCMB to live *M*. *leprae* down regulates the specific cellular immune response against the pathogen. Moreover, our data suggest that BCG vaccination of HCMB induces activation of T cell clones, likely through “trained immunity”, that recognize *M*. *leprae* specific antigens not shared with BCG as an additional protective mechanism besides the expected boost in cell-mediated immunity by BCG homologues of *M*. *leprae* antigens.

## Author summary

Leprosy remains a global public health issue with an annual new case detection of approximately 200,000–250,000 patients. The current study targets leprosy patient contacts, who constitute the group of individuals at highest risk of developing the disease. Treatment of the index case (patient) and BCG vaccination of his/her contacts are among the measures known to decrease the risk of household leprosy contacts contracting the disease. In the present work, the impact of these two measures on the immune response of contacts to mycobacterial antigens was investigated, showing improvement in the cellular immune response to both specific and shared *M*. *leprae* antigens and an increase in secretion of proinflammatory mediators, which likely explains the protective effect of these measures against leprosy.

## Introduction

Leprosy is a global disease with no efficient means for early diagnosis or prevention. While disease prevalence has dropped significantly over the past three decades, it has not been completely eliminated (averaging between 200,000–250,000 new cases each year) [1]. Leprosy constitutes a public health threat particularly in Brazil in that about 30,000 new cases are reported per annum [2]. Thus, understanding the human immune response to this pathogen remains an important challenge in the development of novel tools for leprosy control.

*Mycobacterium leprae*, the causative agent of leprosy, is a highly infectious obligate intracellular bacterium. Although the vast majority of those exposed to *M*. *leprae* becomes infected, only a small proportion evolves into active disease. Previous work in leprosy and tuberculosis has revealed the major role played by interferon-γ (IFN-γ), an effector cytokine produced by pathogen-specific memory CD4 T cells, in the control of the infection by these intracellular pathogens [3][4].

Leprosy is manifested across a broad spectrum of clinical forms that are determined by the intensity of an individual´s cellular immune response to *M*. *leprae*. The paucibacillary (PB) [polar tuberculoid] (TT), and borderline tuberculoid (BT)] forms of leprosy manifest a contained, self-limited infection with few lesions in which scarce bacilli are detected in consequence of the cellular immune response against *M*. *leprae*. In contrast, the reduced specific cellular immunity in patients with the multibacillary (MB) forms of the disease [lepromatous (LL) and borderline lepromatous (BL)] results in an uncontrolled proliferation of the leprosy bacillus accompanied by many lesions and extensive infiltration in the skin and nerves. MB patients are considered the main source of *M*. *leprae* transmission since they carry a high bacterial load in their skin and are able to shed large numbers of bacteria from their nasal passages at a daily average of 10^7^ viable *M*. *leprae* [5]. Thus, household contacts of MB leprosy patients (HCMB) are at the highest risk of developing leprosy due to their proximity with their index cases and consequent overexposure to *M*. *leprae*. In fact, the risk of illness among HCMB is 8–10 times higher than that of healthy individuals residing in endemic areas but with no domiciliary exposure to MB leprosy [6]. Some studies have shown this risk to be even more elevated among contacts carrying specific antibodies against the phenolic glycolipid-I (PGL-I) antigen [7][8][9], a unique molecule located in the *M*. *leprae* cell wall. Despite the fact that leprosy contacts constitute a group at a high risk to develop leprosy, only about 7% of them will progress to active disease[10][11].

Evidence indicates that persistent exposure to *M*. *leprae* leads to a reduced lymphoproliferation to *M*. *leprae* antigen, which improves as a result of index case treatment [12]. Recent data have confirmed and added to this previous finding, linking persistent exposure to *M*. *leprae* and/or bacillary load in leprosy patients with hyporesponsiveness to *M*. *leprae*-specific antigens. A high level of the *ex vivo* IFN-γ response to *M*. *leprae*-specific peptides was observed in nearly all the exposed healthy individuals. However, there was a progressive reduction in these levels that correlated with the exposure level to *M*. *leprae* infection. The IFN-γ levels were lowest among HCMB when compared to PB household contacts and individuals residing in an hyperendemic leprosy area but with no history of contact with the disease [13].

It is well established that BCG confers protection against leprosy [14][15][16]. Since 1991, the Brazilian Ministry of Health (BMH) has officially recommended that household contacts (HC) of leprosy patients must be revaccinated with BCG to boost the efficacy of the first dose given to newborns as a tuberculosis prophylactic vaccine. The rationale for the use of BCG as a vaccine against leprosy relies on the knowledge that *M*. *leprae* and *M*. *bovis* BCG share many antigens with a high degree of homology [17]. In leprosy HC, the vaccine is administered irrespective of tuberculosis or leprosy skin test results. HC with no BCG scar at all or with only one are vaccinated with BCG. Healthy HC with two BCG scars are not vaccinated [18]. In Brazil, the impact of this policy, assessed in a cohort study of 3536 contacts of 1161 leprosy patients, revealed that the protection conferred by a booster BCG vaccine was 56%. The strain used in Brazil is known as BCG Moreau whose complete genome sequence has recently been deciphered [19].

Although both the BCG vaccination and index case treatment decrease the risk of household contacts contracting leprosy [9], the changes in the immune response induced by these two measures that could possibly explain the resulting protective effect have yet to be investigated in detail. In the present work, a prospective study was conducted to assess the impact of BCG vaccination and index case treatment on the *ex vivo* frequencies of CD4^+^ and CD8^+^ T cells responsive to *M*. *leprae* specific and shared mycobacterial antigens. The patterns of the cytokine/chemokine responses of the peripheral blood mononuclear cells (PBMC) stimulated *in vitro* with the same antigens were also studied. A better understanding of the mechanisms responsible for the BCG-conferred protection against leprosy and the down modulation of the *M*. *leprae*-specific immune response by the overexposure to live bacteria among leprosy contacts could contribute to defining the biomarkers of protective immunity against mycobacteria and lead to the development of better and more effective vaccines.

## Methods

### Ethics statement

Ethical approval of this study was obtained from the Oswaldo Cruz Foundation Committee for Ethics in Research. Participants were informed about the study objectives together with the required amount and kind of samples. Written and informed consent was obtained from study participants before enrollment.

### Recruitment of household contacts

A total of 16 household contacts of multibacillary leprosy patients (HCMB) with an average age of 37 ± 13 years, consisting of 11 females and 5 males under surveillance at the Souza Araújo (Fiocruz) Outpatient Clinic (Reference Center for Leprosy Diagnosis and Treatment, Oswaldo Cruz Foundation (FIOCRUZ), in Rio de Janeiro, RJ, Brazil, were enrolled in the study. All participants resided in the State of Rio de Janeiro, which, in 2013, had a new leprosy case detection rate of 7.36/100 000 individuals. HCMB were assessed for the presence of a BCG scar and the absence of clinical signs and symptoms of leprosy via routine examinations (dermatological, neurological, and/or physiotherapeutic assessments). Serology was carried out via ML Flow or ELISA for detection of anti-PGL-I IgM. HCMB received 0.1 ml of BCG (Ataulpho de Paiva Foundation, Rio de Janeiro, RJ, Brazil) intra-dermally in the deltoid region of the arm at about one-third down the upper arm over the insertion of the deltoid muscle [20].

### *M*. *leprae* whole-cell sonicate and synthetic peptides

Irradiated armadillo-derived *M*. *leprae* whole cells were probe sonicated with a Sanyo sonicator to 95% breakage. This material was provided by the NIH/NIAID ‘‘Leprosy Research Support” Contract N01 AI-25469 from Colorado State University (USA) (These reagents are now available through the Biodefense and Emerging Infections Research Resources Repository listed at https://www.beiresources.org/Catalog/antigen/NR-19329.aspx

Synthetic peptides of 15 amino acids corresponding to class II *M*. *leprae*-specific epitopes were produced by Peptide 2.0 Inc. (Chantilly, VA, USA). For the present study, 13 HLA class II-restricted, *M*. *leprae*–specific peptides [p38, p51, p56, p59, p65, p67, p70, p71, p88, p91, and p92 (10 μg/ml each)] of 15 amino acids were combined and used as a pool for culture stimulation. These peptides correspond to *M*. *leprae* epitopes that share any or low similarity with BCG and are referred to as *M*. *leprae* specific in this study. They were previously tested for the induction of IFN-γ- in the PBMC of leprosy patients and their contacts, and of healthy controls in both endemic and non-endemic areas for leprosy. Only responses in leprosy patients and in healthy individuals exposed to *M*. *leprae* were observed, indicating that they specifically detect individuals infected with *M*. *leprae* [21].

### PBMC isolation and stimulation

Peripheral blood mononuclear cells (PBMCs) obtained from heparinized blood were isolated using Ficoll-Paque (GE Healthcare Life Sciences Pittsburgh, PA, USA) and resuspended in AIM V medium (Invitrogen, Grand Island, NY, USA) supplemented with 100 U/ml penicillin, 100 mg/ml streptomycin, and 2 mM L-glutamine (Sigma Chemical, St. Louis, MO, USA). PBMC from each individual were seeded at 2 x 10^6^ cells per well and stimulated with anti-CD28 [1μg/ml] and anti-CD49d monoclonal antibodies [1μg/ml] (BioLegend, San Diego, CA, USA) plus armadillo-derived *M*. *leprae* cell sonicate (20 μg/ml), M. *leprae*-specific peptides [10μg/ml each], or staphylococcal enterotoxin B (SEB, 1μg/ml; Sigma) for a period of 6 hrs before the flow cytometry analysis. Protein transport inhibitor (Brefeldin A—BD, San Jose, CA, USA) was added during the last hour of incubation. In parallel, 2 x 10^5^ cells were seeded in 96-well plates and stimulated with the *M*. *leprae* cell sonicate (20 μg/ml), *M*. *leprae*-specific peptides (10 μg/ml each), or SEB (1μg/ml) for 5 days. Supernatants were collected and immediately stored at -20°C until use.

### Flow cytometry

After a 6-hr culture, the cell populations were stained with a live/dead fixable violet dead cell stain kit (Invitrogen, Carlsbad, CA, USA) for distinction of dead cells according to the manufacturer's instructions, and the following monoclonal antibodies were used: anti-hCD3-V500, anti-hCD4-PerCP, anti-hCD8-Alexa 700, anti-hCD69-APCCy-7, anti-hCD45RO-PECY7, or CD45RA-PECY7 / anti-hCD62L (BioLegend, San Diego, CA and BD biosciences San Jose, CA, EUA). Flow cytometric analysis was performed using a FACSAria IIu flow cytometer (BD Biosciences) and the FlowJo software version 7.5 (FlowJo LLC, Ashland, OR, USA).

### Measurement of inflammatory mediators

A multiplex biometric immunoassay containing fluorescent-dyed microspheres conjugated with a monoclonal-specific antibody for a target protein was used for measurement of inflammatory mediators according to the manufacturer´s instructions (Bio-Plex Pro Human Cytokine 17-plex Assay; Bio-Rad Inc., Hercules, CA, USA). The mediators measured were: IL-1β, IL-2, IL-4, IL-5, IL-6, IL-7, IL-8, IL-10, IL-12 (p70), IL-13, IL-17, G-CSF, GM-CSF, MCP-, MIP-1β, IFN-γ, and TNF mediator levels were determined by a multiplex assay reader from the Luminex Instrumentation System (Bio-Plex Workstation from Bio-Rad Laboratories, Inc.). Analyte concentration was estimated according to the standard curve using the Bio-Plex Manager software provided by the manufacturer. Values of unstimulated cultures were discounted from all stimuli.

### Statistical analysis

Graphs were created using the GraphPad Prism 5 software (GraphPad Software, La Jolla, CA, USA), and the paired nonparametric Wilcoxon test was utilized to perform statistical analyses. A *p* value of 5% or less was considered significant. Medians were compared by either the Wilcox Signed Rank Test (for paired groups) or the Mann-Whitney Test (for unpaired groups). For association analyses, Spearman's Rank Correlations Coefficient with the Bonferroni correction of the family-wise error rate was adopted. Multivariate principal component analysis (PCA) was performed for dimension reduction and visualization using the R version software 3.1.2.[22][23]

## Results

### Epidemiological data and study design

**Fig 1:**
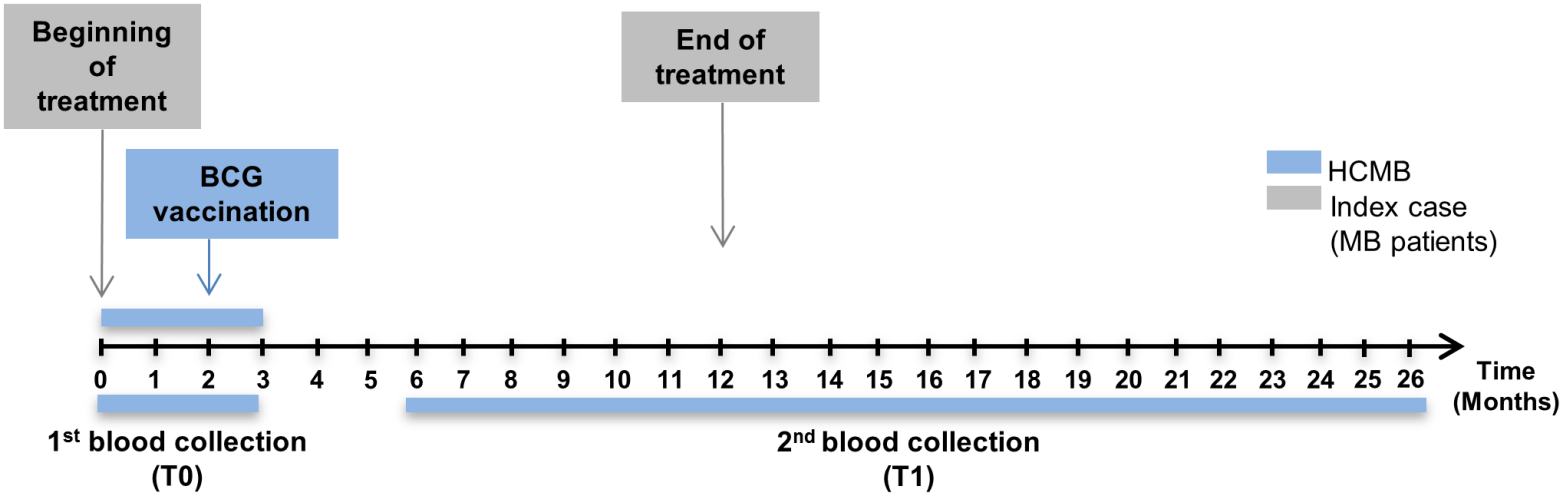
Study design. HCMB were enrolled during a period of 0–3 months after the beginning of their index case treatment. The first blood collection was performed prior to BCG vaccination according to the presence/absence of a BCG scar (T0). After an interval of 6 to 26 months from chemotherapy onset of the index case, the same individuals were asked to provide new peripheral blood samples (T1).

A prospective study was conducted in HCMB to evaluate the impact of BCG vaccination and interruption of persistent exposure to live *M*. *leprae* by treating their index case on the *ex vivo* immune response to the pathogen. Blood was collected from the HCMB for comparative evaluation at the outset of the MB leprosy index case treatment prior to BCG contact vaccination (T0) and at least 6 months after the beginning of treatment (T1; Fig 1). The BCG vaccine was administered to contacts according to their vaccination history. As shown in Table 1, 13 out of the 16 HCMB were vaccinated at T0, 5 of whom received their first BCG dose and 8, a second dose due to their BCG scar. Three did not receive the vaccine at T0; 2 had previously received two doses (presence of 2 BCG scars), and the third, with no BCG scar, was suspected of leprosy and not vaccinated. However, this particular HCMB did not become ill during follow up. Ten HCMB were found to be seropositive to anti-PGL-I. However, no correlation between seropositivity to PGL1 and index case BI was observed (Table 1).

**Table 1:** Epidemiological data of MB leprosy patient household contacts.

Contact Identification	Age (years) at first examination	Neonatal BCG vaccination	BCG vaccination at T0	IgM antibodies against PGL-I	Clinical form of the index case / LBI	Time interval between first and second evaluation (months)
1	52	no	yes	negative	BL / 4.8	22 months
2	36	no	yes	negative	BL / 3.6	6 months
3	41	no	yes	positive	BL / 3.6	6 months
4	52	no	yes	negative	LL / 5.9	6 months
5	29	no	yes	negative	LL / 5.9	12 months
6	41	yes	yes	negative	LL / 5.9	26 months
7	25	yes	yes	negative	LL / 3.8	26 months
8	46	yes	yes	positive	LL / 4.8	12 months
9	63	yes	yes	negative	BL / 3.8	12 months
10	19	yes	yes	positive	LL / 5.9	20 months
11	48	yes	yes	positive	LL / 5.9	10 months
12	26	yes	yes	negative	LL / 4.8	17 months
13	38	yes	yes	positive	BL / 2.0	6 months
14	43	no	no	positive	BL / 4.8	21 months
15	19	yes	no	negative	BL / 3.6	6 months
16	20	yes	no	negative	LL / 5.9	6 months

Abbreviations: LL, Lepromatous Lepromatous; BL, Borderline Lepromatous; LBI, logarithmic bacillary index of skin lesion;

T0 indicates the beginning of index case treatment and prior to BCG vaccination; and T1 indicates the end of index case treatment and after BCG vaccination.

### Index case treatment and BCG vaccination increase *ex vivo* frequencies of CD4^+^ cells specific for *M*. *leprae*-specific antigens

**Fig 2:**
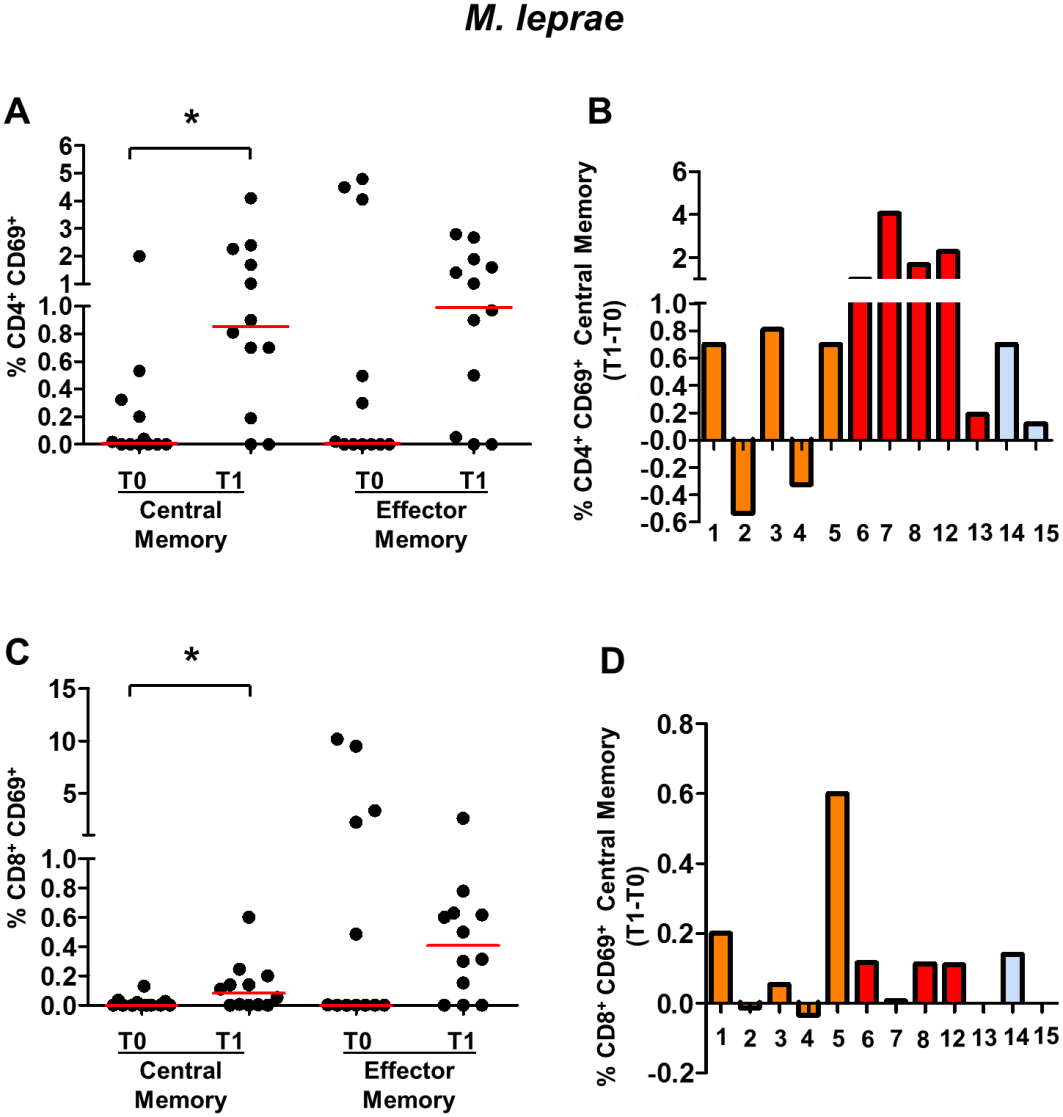
Frequencies of memory CD4^+^ and CD8^+^ T cells responsive (CD69^+^) to *M*. *leprae* cell sonicates in household contacts of multibacillary leprosy patients (HCMB). Frequencies of central and effector memory CD4^+^(A) and CD8^+^(C) T cells expressing CD69 in response to 6-hour *in vitro* stimulation with *M*. *leprae* cell sonicate at T0 and T1. Each black circle represents one HCMB. The red lines represent the median values at the different time points. Individual lymphocyte frequencies of central memory CD4^+^ CD69^+^ T cells (B) and central memory CD8^+^ CD69^+^ T cells (D) after index case treatment and BCG vaccination of the HCMB. Each bar represents a single contact identified by the number under the bar. Orange bars represent contacts who received the first BCG dose at T0; red bars represent the HCMB who received a second BCG dose at T0; and the blue bars represent the HCMB who were not BCG vaccinated at T0. n = 12. * p<0.05.

Exposure to pathogens is followed by the generation and persistence of memory T cells, which can provide long-lasting protection against these same pathogens (25). The impact on index case treatment and BCG vaccination in *M*. *leprae*-responsive T cell frequencies in peripheral blood was evaluated by detecting the T cells expressing the early activation antigen CD69 (CD69^+^) in response to short-term *in vitro* stimulation with *M*. *leprae*-specific antigens. To analyze the frequency of central memory CD4^+^ T cells (TCM) and effector memory CD4^+^ T cells (TEM) responsive to *M*. *leprae*, the gate strategy shown in S1 Fig was applied. This analysis was performed in 12 HCMB. PBMC from this group were stimulated with two antigen preparations: i) a *M*. *leprae* cell sonicate, which is a complex antigen mixture that is mostly shared with BCG; and ii) a pool of synthetic *M*. *leprae*-specific peptides corresponding to HLA class II-restricted epitopes. Regardless of their BCG vaccination status, almost all HCMB showed an increased frequency of CD4^+^ TCM responsive to the *M*. *leprae* cell sonicate at T1 (10 out of the 12 contacts) (Fig 2A and 2B). In addition, effector memory CD4^+^ TEM responsive to *M*. *leprae* frequencies increased in 8 out of 12 HCMB at T1 (S1 Fig). Moreover, when analyzing CD8 T cell frequencies, a significant increase was detected in the CD8^+^ TCM responsive to *M*. *leprae* at T1 (7 out of the 12 contacts) (Fig 2C and 2D). Interestingly, the increase in CD4 and CD8 TCM frequencies was observed even in those HCMB in which the interval between the first and second evaluations was 20–26 months (HCMB# 1, 6, 7 14). Of note, even HCMB who were not BCG vaccinated at T0 (HCMB#14 and 16) showed increased frequencies of the CD4^+^ T and CD8^+^ T cells responsive to *M*. *leprae* at T1. No correlation was seen between individual frequencies to *M*. *leprae*-responsive T cells and seropositivity to PGL-I among HCMB at any time point.

**Fig 3:**
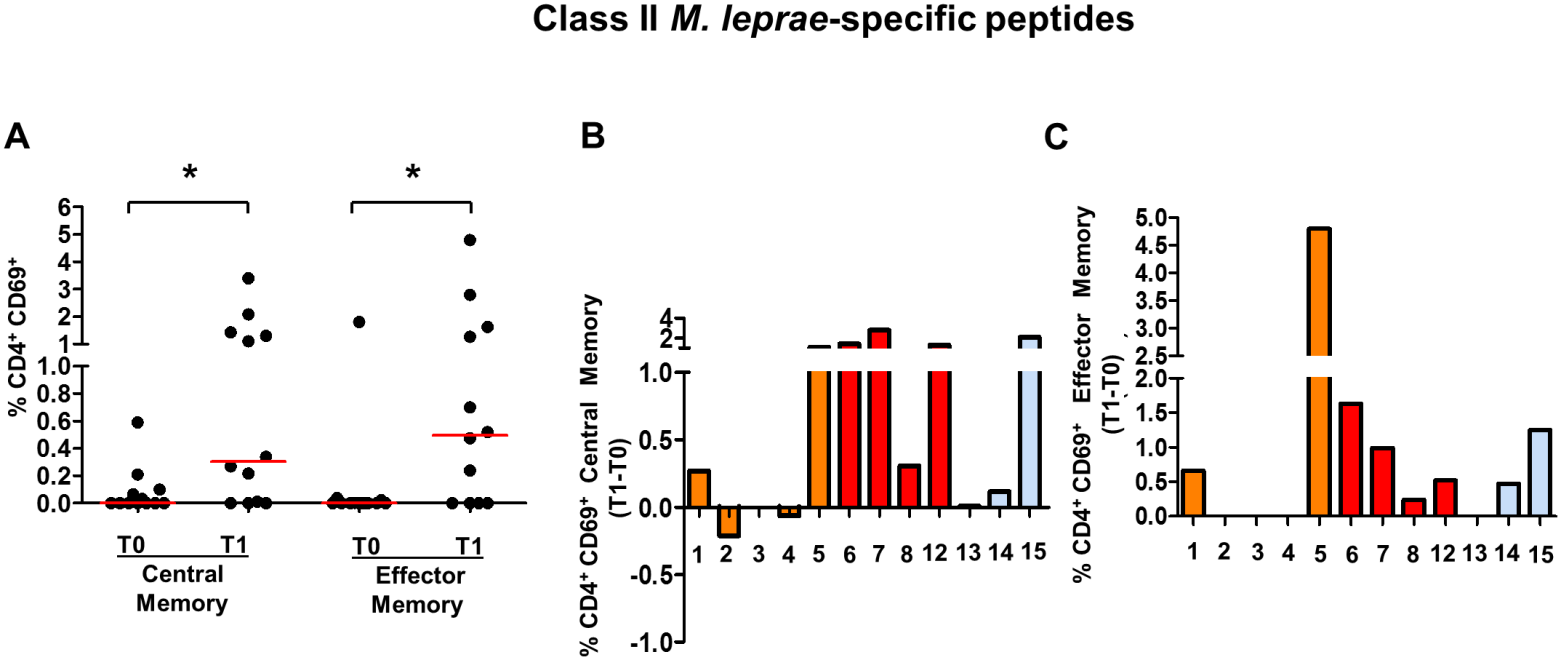
Frequencies of CD4^+^ T cells responsive (CD69^+^) to HLA Class II-restricted *M*. *leprae*- specific epitopes in household contacts of multibacillary leprosy patients (HCMB). (A) Frequencies of central and effector memory CD4^+^ T cells expressing CD69 in response to 6-hr *in vitro* stimulation with a pool of HLA class II-restricted synthetic peptides derived from *M*. *leprae* specific epitopes (15-mers) at T0 and T1. Each black circle represents one HCMB. The red lines represent the median values for the different time points. Changes in the Individual frequencies of central (B) and effector memory T cells (C) responsive to *M*. *leprae* specific epitopes are shown. Each bar represents a single contact identified by the number under the bar. Orange bars represent the contacts who received the first BCG dose at T0; the red bar represents the HCMB who received a second BCG dose at T0; and the blue bars represent the HCMB who was not vaccinated at T0. n = 12. * p<0.05.

The *ex vivo* frequencies of CD4^+^ T cells in the *M*. *leprae*-specific peptides are shown in Fig 3. It is noteworthy that a significant increase in TCM and TEM CD4^+^ T cell frequencies in response to the *M*. *leprae* peptides was in evidence at T1. Interestingly, HCMB who did not receive a BCG vaccination at T0 (HCMB#14 and 16) also showed increased CD4^+^ TCM (Fig 3B) and TEM frequencies (Fig 3C) in response to *M*. *leprae*-specific peptides at T1. There was no difference in response between the positive or negative HCMB to PGL-I antibodies; and the frequencies of peptide-pool specific CD8^+^ T cells were below the detection limit.

### Index case treatment and BCG vaccination increase the *in vitro* levels of pro-inflammatory mediators in response to mycobacterial antigens

**Fig 4:**
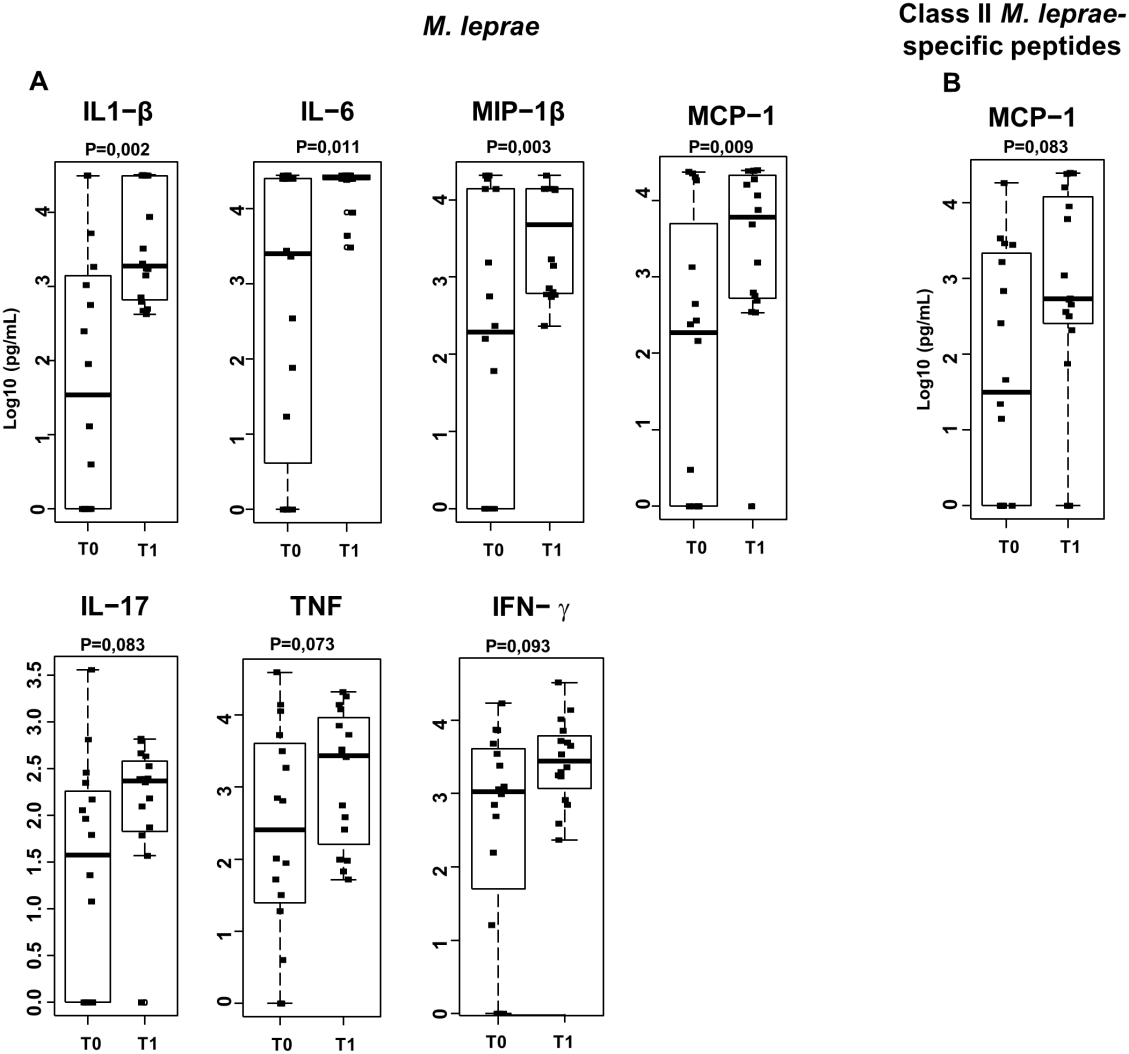
Effector molecule levels increase in response to *M*. *leprae* after index case treatment and BCG vaccination of household contacts of MB leprosy patients (HCMB). Levels of effector molecules in supernatants of 5-day cultures stimulated with ML (A) or a pool of HLA Class II-restricted ML-specific synthetic peptides (B) were evaluated by multiplex assays in supernatants of 5-day cultures of peripheral blood leukocytes of HCMB before (T0) and after (T1) treatment of their index cases and BCG vaccination. Box plots show median, interquartile range, sample minimum, and maximum levels. Dots represent individual donors. n = 16.

Next, comparisons were made among the cytokine, chemokine, and growth factor levels secreted *in vitro* by PBMC stimulated with the *M*. *leprae* cell sonicate or *M*. *leprae*-specific peptides at T0 and T1. In the unstimulated cultures, the levels of these biomarkers were either below or, in a few cases, just above the detection limit. All the individuals responded well when their cells were cultured in the presence of the superantigen staphylococcal enterotoxin B used as a positive control. Among the 17 mediators measured by a multiplex assay, a significant increase was observed at T1 in the proinflammatory cytokines IL-1 β and IL-6 and the chemokines MCP-1 and MIP-1β. Likewise, a tendency toward higher levels of TNF (p = 0.073), IL-17 (p = 0.083), and IFN- γ (P = 0.093) (Fig 4A) was demonstrated. Among the inflammatory mediators, IL-17 and IL-1 β showed a positive correlation (R = 0.7 and p = 0.001) in response to *M*. *leprae* at T0 and T1 and IL-8 production levels in all individuals were above the upper detection limit. Individual behavior of each HCMB in terms of secretion of these mediators is displayed in S2 Fig. HCMB, whether vaccinated or not at T0 (HCMB#14, 15 and 16), showed increased levels of inflammatory mediators in response to *M*. *leprae* at T1. The increment in inflammatory mediators was observed even in those HCMB in which the interval between the first and second evaluations was 20–26 months (HCMB# 1, 6, 7, 10, 14). It is probable that a more consistent increase along with higher levels of mediator production occurred among the contacts receiving a second BCG dose at T0. Nonetheless, the differences did not reach the level of statistical significance (S2 Fig). There was no difference in response between HCMB anti-PGL-I positive and negative individuals. In cultures stimulated with *M*. *leprae*-specific peptides, only MCP-1 levels were suggestive of a more robust response (Fig 4B).

**Fig 5:**
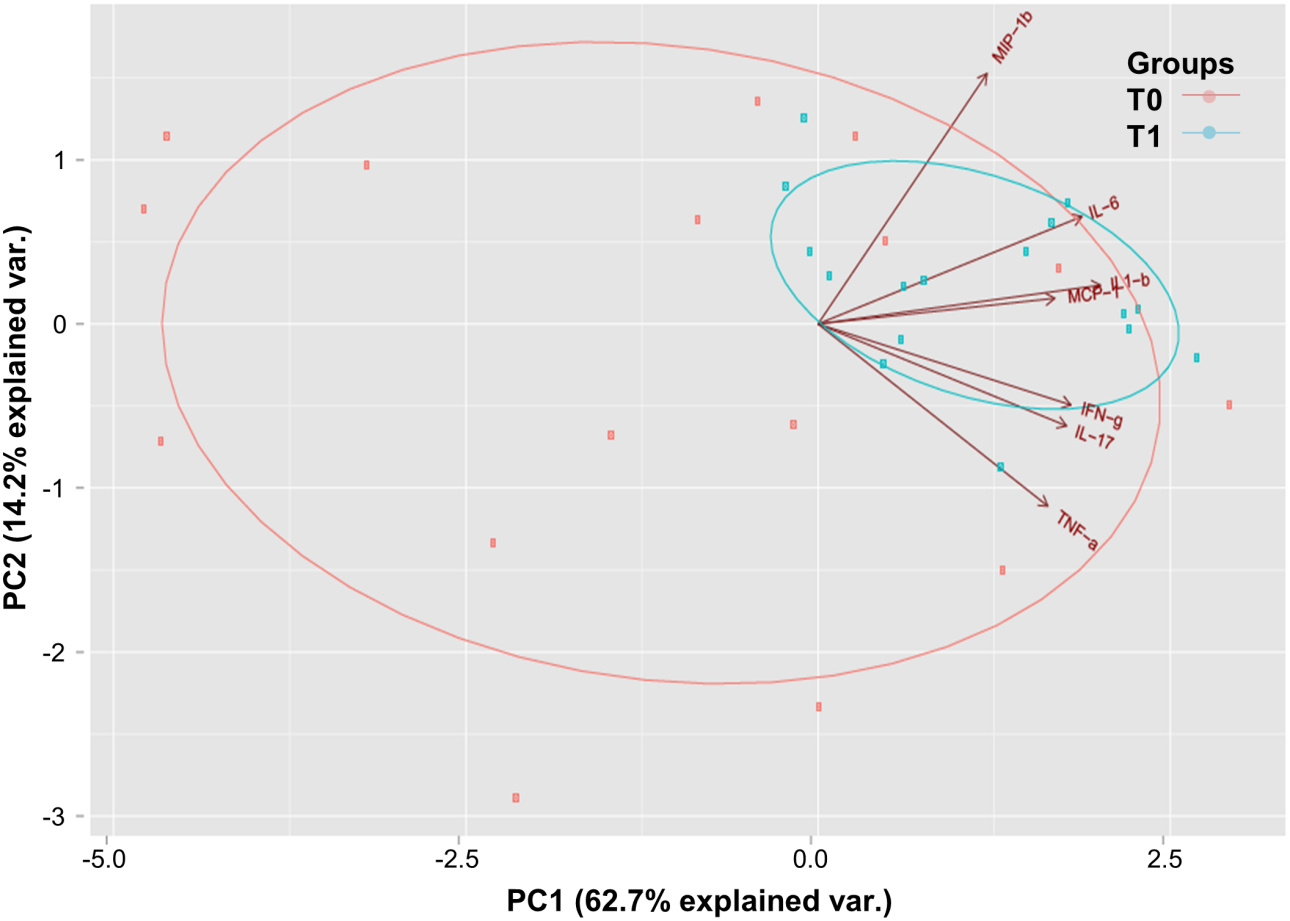
Pattern of cytokine response to *M*. *leprae* becomes more homogeneous after BCG vaccination and index case treatment. Principal component analysis scored values for components 1 and 2. Longer vectors (in brown) indicate the cytokines that responded to *M*. *leprae*. T0 samples are in salmon (n = 16) and T1 in blue (n = 16).

It was then decided to evaluate if the increased levels of MCP-1, IL-1β, IL-17, IL -6, IFN- γ, MIP-1β, and TNF observed at T1 in response to *M*. *leprae* would differentiate T0 from T1 when analyzed simultaneously. In the principal component analysis (PCA), 76.9% of the total variation in response to the 7 cytokines could be narrowed down to 2 components. The first component accounted for a full 62% of the total variation, coming close to corresponding to the average standardized log response to IL-1β, IL-6, IFN-γ, IL17, MCP-1, TNF, and MIP-1β. The second component was independent of the first, totaling 14% of the remaining variation, with a close approximation to the average standardized log response to MIP-1β and IL-6 (Table 2). Together, these cytokines did not completely differentiate T0 from T1. However, a greater heterogeneity in response at T0 was found in contrast to the homogeneity in response at T1 (Fig 5), suggesting a trend toward separation.

**Table 2:** Principal component analysis.

Component	Eigenvalue	Difference	Proportion	Cumulative
Component 1	4.25	3.29	62.73	62.73
Component 2	0.96	0.44	14.18	76.91
Component 3	0.52	0.10	7.73	84.64
Component 4	0.43	0.07	6.27	90.91
Component 5	0.36	0.19	5.27	96.18
Component 6	0.17	0.08	2.48	98.66
Component 7	0.09	NA	1.34	100.00
**Variable**	**Component 1**	**Component 2**		
IFN-γ	0.39	-0.23		
IL-17	0.39	-0.29		
IL-1β	0.44	0.11		
IL-6	0.41	0.30		
MCP-1	0.37	0.07		
MIP-1β	0.26	0.70		
TNF	0.36	-0.51		

Principal component analysis of the *ex vivo* response to *M*. *leprae* at T0 and T1 (cytokine levels in *M*. *leprae*-stimulated 5-day culture supernatants of peripheral blood leukocytes). NA, not applicable.

## Discussion

The current study targeted HCMB, the group of individuals exposed to leprosy at the highest risk of developing active disease. Two factors are known to decrease the risk of disease among HCMB: i) treatment of the index case (patient) decreases exposure to live *M*. *leprae*[24]; and ii) BCG vaccination[9]. In the present prospective study, the impact of these two factors on the HCMB immune response to mycobacterial antigens was investigated. The frequencies of the peripheral blood memory T cells responsive to *M*. *leprae* and the levels of inflammatory mediators produced in *M*. *leprae*-stimulated cultures among HCMB were evaluated before and after BCG vaccination and treatment of their index cases.

Our findings indicate changes in the HCMB immune response to mycobacterial antigens that could account for their improved resistance to developing leprosy, as follows: i) an increase in the frequencies of memory CD4 and CD8 T cells responsive to the *M*. *leprae* whole-cell sonicate; ii) higher frequencies of CD4^+^ T cells that recognize *M*. *leprae*-specific peptides; and iii) higher production levels of the inflammatory mediators IL1-β, IL-6, IL-17, TNF, IFN-γ, MIP1-β, and MCP-1 by PBMCs in response to mycobacterial antigens. Of note, the improved response against *M*. *leprae* antigens in HCMB seems to be a long-lasting effect, since it was observed even after two years of follow up. Moreover, an increment of these parameters was observed even among contacts that did not receive a BCG vaccine at T0, suggesting that reduced exposure to live *M*. *leprae* in consequence of index cases treatment constitutes an important element in the enhanced immune response witnessed in these individuals.

Interestingly, in HCMB, an increment in both *M*. *leprae*-specific memory CD4^+^ and CD8^+^ T cell frequencies was observed at T1. CD4 and CD8 T cells have been implicated in the protective immune response against mycobacteria [25] and might, therefore, account for the improvement in their protective response against leprosy. Moreover, the borderline increment in their IFN-γ and IL-17 levels in response to *M*. *leprae* observed at T1 points to the activation of Th1 and Th17 T cell subsets, previously shown to be induced by BCG vaccination [26] and implicated in the protection against mycobacteria [27].

An important finding in the present study was the increase in CD4 T cells specific for *M*. *leprae* specific epitopes not found in BCG. This result is in agreement with a previous study in which increased levels of IFN-γ were observed in response to MMPI, a *M*. *leprae* antigen not shared with BCG, subsequent to contact vaccination [28]. This could be the result of the well-known, non-specific “adjuvant” effect of BCG on the immune response recently shown to be mediated by innate immune cell epigenetic modifications, referred to as “trained immunity” [29]. Indeed, the increase in TNF, IL-1β, IL-6, MCP-1, and MIP-1β mediators typically produced by monocytes supports the idea of BCG-induced “trained immunity” as a Th1/Th17 heterologous mediating mechanism of immune activation favoring disease protection of HC of leprosy patients. Other reports have shown that most of these mediators are induced by BCG vaccination [30][31][32]. The phenotypic modification of innate immune cells by BCG has been shown to last for at least one year after vaccination [26], an interval compatible with the 6–26 month follow-up adopted in the present study. This is also in line with the long-term BCG protection effect against leprosy previously described [33].

The likely activation of Th1/Th17 T cell populations in conjunction with the simultaneous increment of the inflammatory cytokines/chemokines (TNF, IL-1β, IL-6, MCP-1, and MIP-1β) in response to BCG could explain the onset of paucibacillary leprosy (PB) in a small percentage of leprosy contacts after vaccination[28][10][34][35]. A similar explanation could be applied to the incidence of relatively high numbers of patients with Type 1 reactions among the previously asymptomatic contacts who developed leprosy soon after BCG[35]. According to Bagshawe et al., [34] the manifestation of PB leprosy after BCG vaccination reflects the potential of this vaccine to accelerate evolution to clinical disease in individuals who were infected prior to or immediately after vaccination. In line with this hypothesis, Duppre et al.[10] found that, for the most part, vaccinated contacts contracted leprosy from MB index cases, suggesting that subclinical infection may become overt due to vaccination-induced immune response activation. Moreover, Duppre et al.[9] reported that the incidence of PB leprosy was highest during the first year of follow-up for the PGL-I-positive vaccinated contacts in comparison with the PGL-I negative ones. In the present study, however, no contact developed leprosy post-vaccination during the 3-year follow-up. Likewise, there was no correlation between the presence of PGL-I antibodies and the specific immune response levels observed at T1. In a future study, it may be advisable to increase the sample size to more thoroughly evaluate the impact of anti-PGLI in the immune response to *M*. *leprae* among leprosy patient contacts.

Importantly, an increased cellular immune response to both specific-and-shared *M*. *leprae* antigens was also detected in the 3 contacts who did not receive BCG at T0. This observation is consistent with a previous finding indicating an increase in the PBMC proliferative response to *M*. *leprae*-antigenic preparations among HCMB a full 6 months after initiating index case treatment [12]. The analysis of the immune response to *M*. *leprae* specific antigens of healthy individuals with no history of household contact with leprosy patients, but living in a hyperendemic area for leprosy in Brazil, found high-level IFN-γ responses ex vivo to *M*. *leprae* in all the evaluated individuals from this group. In the same investigation, we observed a progressive reduction in IFN-γ levels with increase of persistent exposure to *M*. *leprae* in asymptomatic infected individuals and leprosy patients [13]. Altogether, these findings support the hypothesis that the continuous exposure to live *M*. *leprae* induces down regulation of the cellular effector immune response against the pathogen and that this effect is reversed upon treatment of the index case. This hypothesis is also supported by data indicating that leprosy incidence decreases significantly among household contacts after three years of index case treatment [34]. It is also known that, even among household contacts, only a small proportion of exposed individuals eventually develop active disease [11]. Overall, the sum of these observations suggests that after the initial infection, there are other yet unknown steps involved in the evolving pathogenesis of leprosy.

Data accrued from previous and the present investigations showing enhancement of *ex vivo* cell-immunity parameters against *M*. *leprae* among HCMB after index case treatment give weight to the hypothesis that persistent exposure may facilitate the evolution of the infection to active disease by inhibiting the effector response in contacts. The negative modulation of the effector immune response to *M*. *leprae*, as a result of continuous and prolonged stimulation of the immune system by the pathogen eliminated by the index case, is a possible explanation for the known high risk of HCMB to evolve from latent infection to the active disease [15][36].

*M*. *leprae*-specific regulatory T cells (Treg) are a potential cause for this down regulation of the effector immune response seen in HCMB. The recent observation of in vitro inhibition of immune response to *M*. *leprae* in lepromatous leprosy by cells with Treg phenotypic characteristics supports this hypothesis. The continuous exposure of the airways immune system of the HCMB to the live *M*. *leprae* aerosols expelled by the MB leprosy patients may create conditions that favor differentiation of *M*. *leprae*-specific Tregs, perhaps by sharing some of the mechanisms inhibiting effector T cell generation in response to environmental antigens and normal microbiome [37].

Tregs have been implicated in the pathogenesis of cancer, autoimmune and infectious diseases as well as allergies. Therapeutic intervention in the Treg function has been successful in some situations, and could, together with index case treatment, be a target in the development of new and improved vaccination strategies for leprosy prevention in populations heavily exposed to leprosy. A deeper understanding of the mechanisms involved in the negative modulation of the immune response experienced by individuals persistently exposed to *M*. *leprae* may contribute to designing tools to more reliably identify infected individuals before there are any clinical manifestations of the disease, which would be a significant contribution toward interrupting the chain of transmission.

To our knowledge, this is the first study demonstrating that index case treatment and/or BCG vaccination of HCMB induce activation of T cell clones that recognize *M*. *leprae* specific epitopes not shared with BCG. This activation may at least partially explain the well-known protective effect of these measures against disease progression in HCMB

## Supporting information

S1 Fig Gates strategy for analysis. After distinction of dead cells by their area and height parameters, singlet cells were selected (A). The lymphocytes were determined via the SSC (side scatter) and FSC (forward scatter) parameters; (B) The CD4^+^CD69^+^ and CD8^+^CD69^+^ central and effector memory T cells were determined with specific antibodies (C and D).
(TIF)

S2 Fig Frequencies of CD4^+^ and CD8^+^ effector memory T cells responsive (CD69^+^) to *M*. *leprae* (T1-T0). Change in the individual frequencies of effector memory CD4^+^ (A) and CD8^+^ (B) T cells expressing CD69 in response to 6-hr *in vitro* stimulation with a *M*. *leprae* sonicate. Each bar represents a single contact identified by the number under the bar. The orange bars represent the contacts that received the first BCG dose at T0; the red bars represent the HCMB that received a second BCG dose at T0; and the blue bar represents the HCMB not BCG vaccinated at T0. n = 12 per group.
(TIF)

S3 Fig Individual production of inflammatory mediators in response to *M*. *leprae*. Supernatants from 5-day cultures of peripheral blood leukocytes stimulated with a *M*. *leprae* sonicate were evaluated using a Multiplex assay at T0 and T1. T0 indicates the beginning of index case treatment and prior to BCG vaccination. T1 indicates after BCG vaccination and treatment of the index case. Each bar represents a single HCMB with number identification. The orange bars represent the contacts that received the first BCG dose at T0; the red bars identify the HCMB that received a second BCG dose at T0; and the blue bars, the HCMB that was not BCG vaccinated at T0. n = 16.
(TIF)
