# Supplementary figures and images for: Interruption of persistent exposure to leprosy combined or not with recent BCG vaccination enhances the response to Mycobacterium leprae specific antigens
Source: PLoS Negl Trop Dis. 2017 May 3;11(5):e0005560. doi: 10.1371/journal.pntd.0005560 (PMC5432189; doi:10.1371/journal.pntd.0005560)

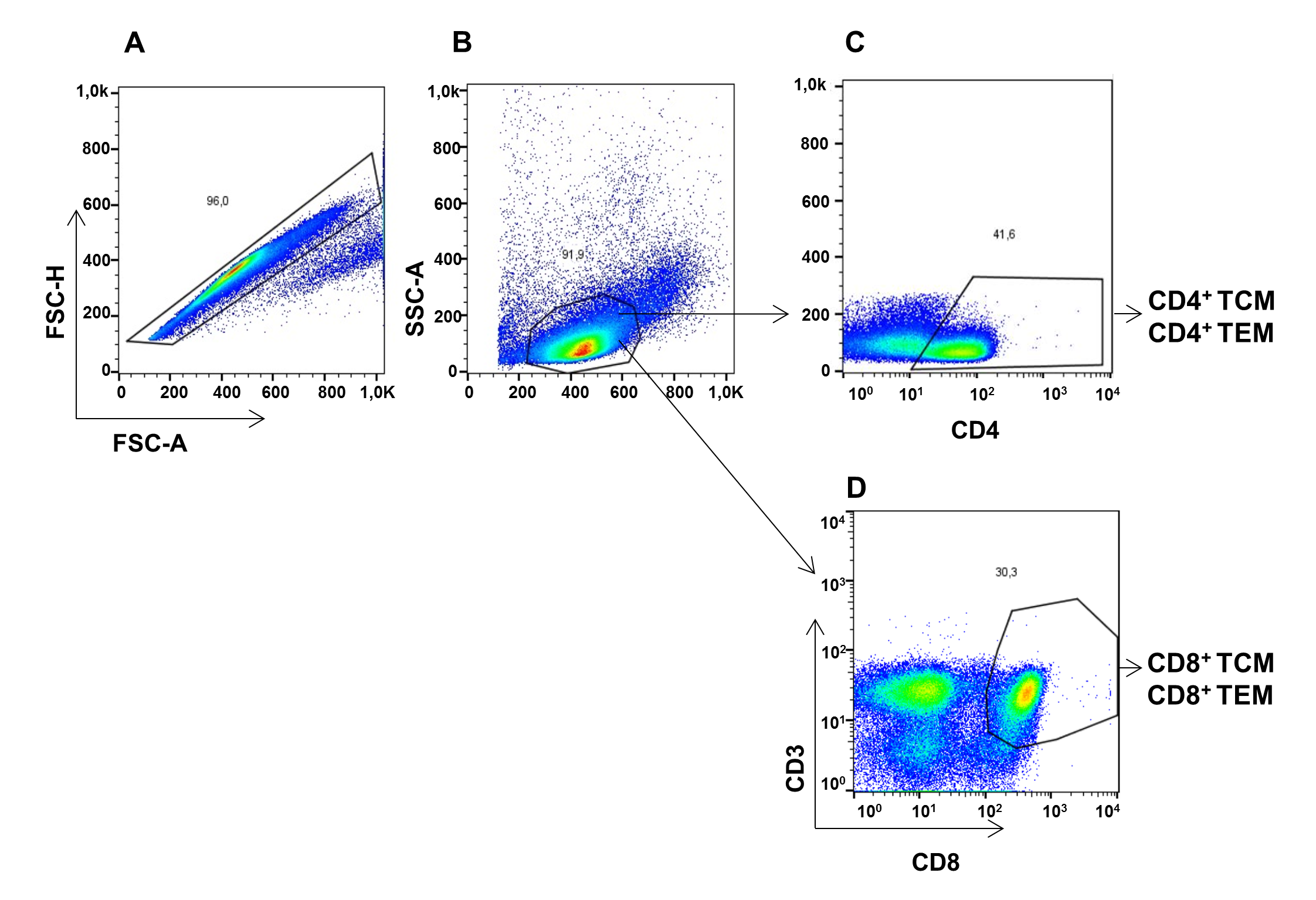

Supplement: S1 Fig — After distinction of dead cells by their area and height parameters, singlet cells were selected (A). The lymphocytes were determined via the SSC (side scatter) and FSC (forward scatter) parameters; (B) The CD4+CD69+ and CD8+CD69+ central and effector memory T cells were determined with specific antibodies (C and D). (TIF) [file pntd.0005560.s001.tif]

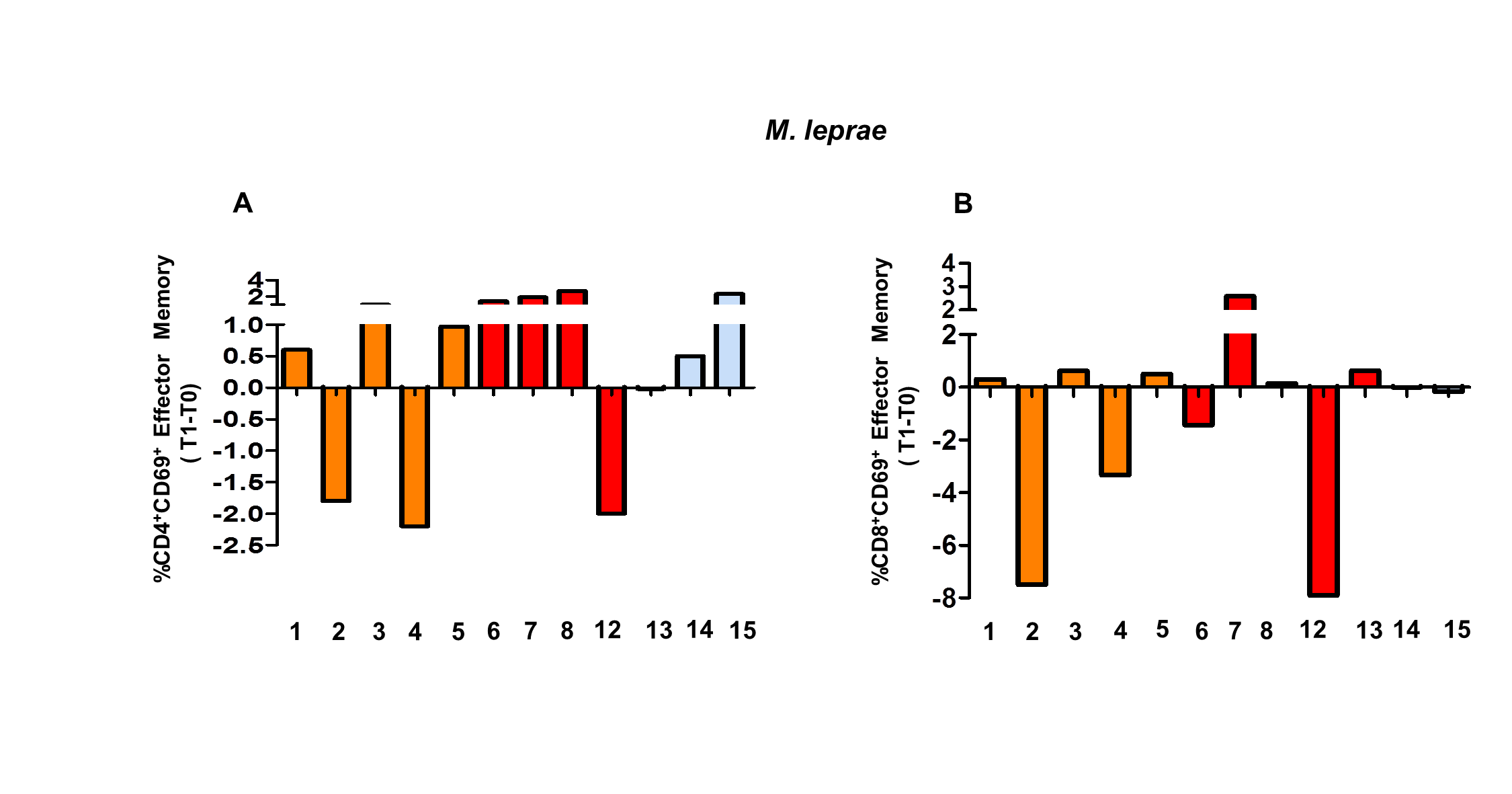

Supplement: S2 Fig — Change in the individual frequencies of effector memory CD4+ (A) and CD8+ (B) T cells expressing CD69 in response to 6-hr in vitro stimulation with a M. leprae sonicate. Each bar represents a single contact identified by the number under the bar. The orange bars represent the contacts that received the first BCG dose at T0; the red bars represent the HCMB that received a second BCG dose at T0; and the blue bar represents the HCMB not BCG vaccinated at T0. n = 12 per group. (TIF) [file pntd.0005560.s002.tif]

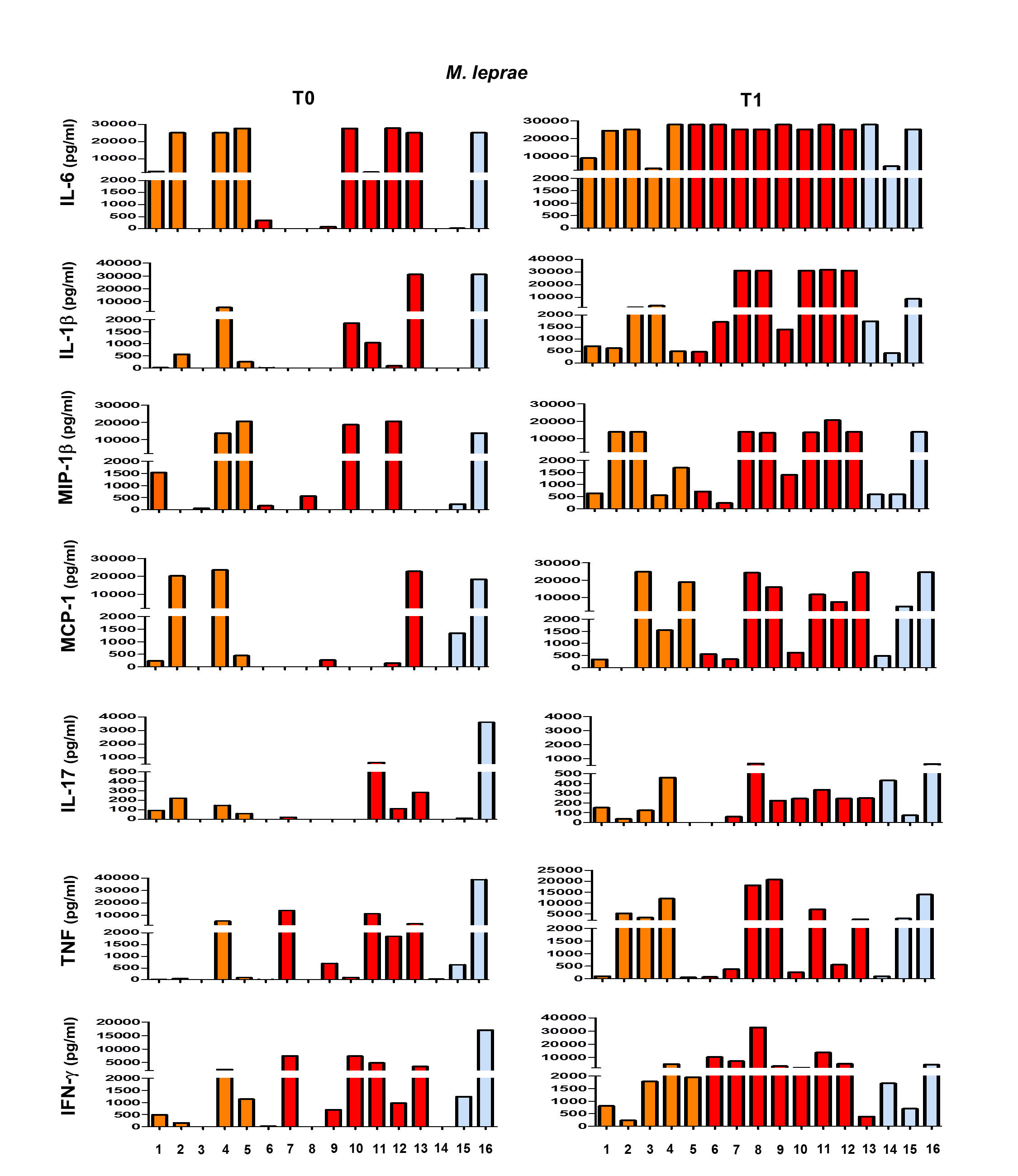

Supplement: S3 Fig — Supernatants from 5-day cultures of peripheral blood leukocytes stimulated with a M. leprae sonicate were evaluated using a Multiplex assay at T0 and T1. T0 indicates the beginning of index case treatment and prior to BCG vaccination. T1 indicates after BCG vaccination and treatment of the index case. Each bar represents a single HCMB with number identification. The orange bars represent the contacts that received the first BCG dose at T0; the red bars identify the HCMB that received a second BCG dose at T0; and the blue bars, the HCMB that was not BCG vaccinated at T0. n = 16. (TIF) [file pntd.0005560.s003.tif]
